# A Review of *Ureaplasma diversum*: A Representative of the *Mollicute* Class Associated With Reproductive and Respiratory Disorders in Cattle

**DOI:** 10.3389/fvets.2021.572171

**Published:** 2021-02-18

**Authors:** Manoel Neres Santos Junior, Nayara Silva de Macêdo Neres, Guilherme Barreto Campos, Bruno Lopes Bastos, Jorge Timenetsky, Lucas Miranda Marques

**Affiliations:** ^1^Department of Biointeraction, Multidisciplinary Institute of Health, Universidade Federal da Bahia, Vitória da Conquista, Brazil; ^2^Department of Microbiology, State University of Santa Cruz (UESC), Ilhéus, Brazil; ^3^Department of Microbiology, Institute of Biomedical Science, University of São Paulo, São Paulo, Brazil

**Keywords:** *Ureaplasma diversum*, Mollicutes, bovine, reproductive tract, respiratory infection

## Abstract

The *Mollicutes* class encompasses wall-less microbes with a reduced genome. They may infect plants, insects, humans, and animals including those on farms and in livestock. *Ureaplasma diversum* is a mollicute associated with decreased reproduction mainly in the conception rate in cattle, as well as weight loss and decreased quality in milk production. Therefore, *U. diversum* infection contributes to important economic losses, mainly in large cattle-producing countries such as the United States, China, Brazil, and India. The characteristics of *Mollicutes*, virulence, and pathogenic variations make it difficult to control their infections. Genomic analysis, prevalence studies, and immunomodulation assays help better understand the pathogenesis of bovine ureaplasma. Here we present the main features of transmission, virulence, immune response, and pathogenesis of *U. diversum* in bovines.

## Mollicutes

**Figure 1:**
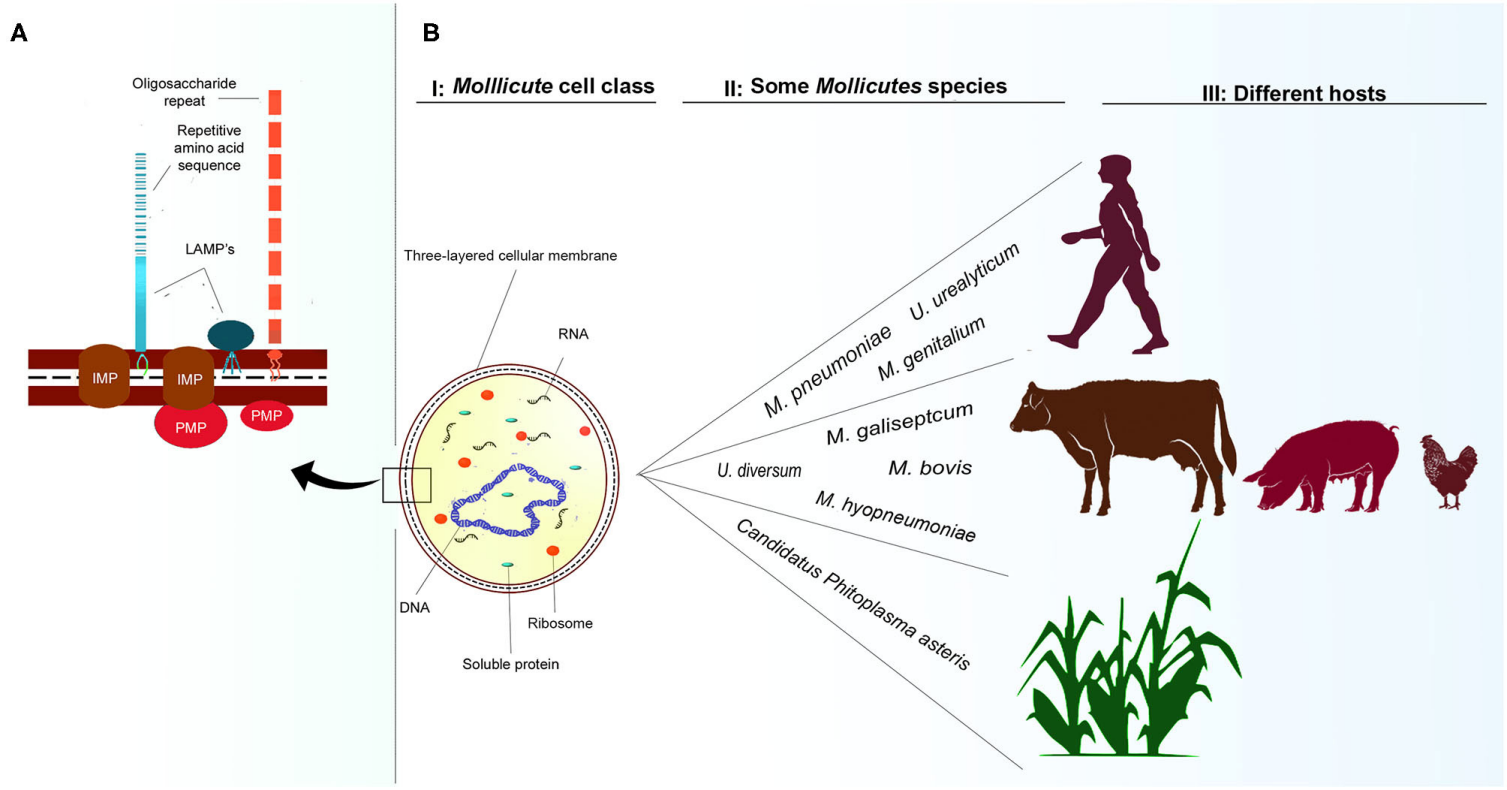
Cellular representation of *Mollicutes* and some hosts. **(A)**: *Mollicutes* cell membrane. Representation of LAMPs anchored to the outer membrane of proteins. IMP, integral membrane protein; LAMPs, lipoproteins; PMP, peripheral membrane protein based on the representation of Chambaud et al. (16). **(BI)**: Single-cell structure of *Mollicutes* demonstrating cell membrane, ribosome, mRNA molecules, DNA molecule, and soluble cytoplasmic proteins. **(BII, BIII)**: some species that infect humans, animals and plants, and some of their respective hosts.

The *Mollicutes* Class (phylum *Tenericutes*) comprises about 200 species. Generally, they are miniscule and considered the smallest self-replicating free-living microorganisms (1–3). Fourteen genera and their representatives are found widely in plants; for example, *Candidatus Phytoplasma asteris, Candidatus Phytoplasma australiense, Candidatus Phytoplasma trifolii*- (4–9); in animals—*Mycoplasma bovis, Mycoplasma gallisepticum, U. diversum* (10–12), and in humans—*Mycoplasma pneumoniae, Mycoplasma genitalium, Ureaplasma parvum, Ureaplasma urealyticum* (13–15). The genera *Mycoplasma, Ureaplasma*, and *Acholeplasma* are known to encompass species inhabiting animals as commensals, saprophytes, or pathogens, as shown in Figures 1BII,III (1, 3, 17).

Lacking a cell wall, these bacteria, essentially have a trilaminated cell membrane with incorporated sterols, ribosomes, and a circular double-stranded DNA molecules, as shown in Figure 1BI (1, 2). They reproduce by binary fission and generate pleomorphic or filamentous and multinucleated forms observed through electron microscopy (18, 19). *Mycoplasmataceae* may be cultured in solid medium and in broth medium. Due to the small size growth in broth, they do not cause turbidity, and bacterial growth is verified an alteration in the pH revealed by the colorimetric change of an acid-base indicator present in the culture medium. In Agar medium, they produce small fried-egg-shaped colonies ranging from 50 to 500 μm (20). Due to their lack of cell wall, Gram staining may allow them to be observed in an optical microscope in a red colored mass.

It is important to highlight that some species produce a polar bleb (21). This structure has been studied mainly for its adherence and the ability of these bacterial cells to travel on glass by caterpillar-like motions (22). In addition, the structure of a complex of organized proteins in *M. pneumoniae*, a human pneumonic species, has been well-described, elucidating how such proteins are able to adhere to and cause pneumonia in animals (22, 23).

The presence of filamentous wire and rod networks indicate the presence of cytoskeleton-like structures and these may also help in maintaining the morphology. Such filaments may also be involved in division and sliding motility in some mycoplasmas (2, 18, 20).

**Figure 2:**
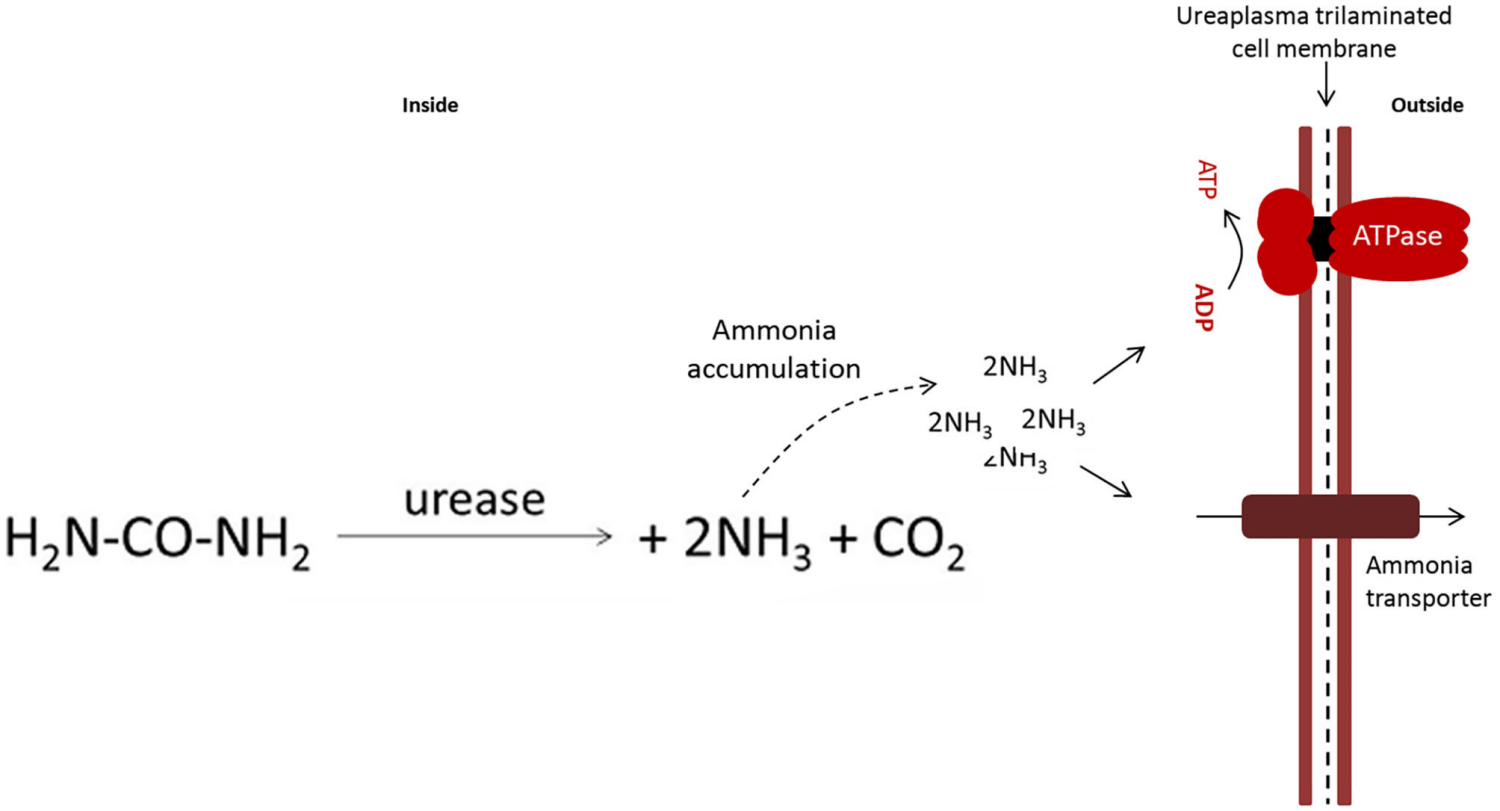
Equation of the conversion of urea to ammonia catalyzed by the enzyme urease present in *U. diversum, b*ased on the model by Marques et al. (28). The accumulation of intracellular ammonia generates a gradient that is used to produce ATP. ATP is used as energy and ammonia causes damage to host tissues.

*Mollicute* metabolism is quite variable. Species that metabolize carbohydrates for energy production are classified as glucose fermenters. Non-fermenting *Mollicutes* lack the arginine dehydrolase pathway to obtain adenosine triphosphate (ATP) (24, 25). In these bacteria, the tricarboxylic acid cycle is incomplete, and they also lack quinones and cytochromes. Consequently, the possibility of effective oxidative phosphorylation as a power generating mechanism is ruled out (2, 26). Some mycoplasmas may rely on fermentative or non-fermentative metabolism depending on the conditions of the microenvironment (27). *Ureaplasma* spp. present a differentiated metabolism; they require urea to generate ATP. Figure 2 and section 3.1 (urea production and modulation in prostaglandin synthesis) provide more details on the production and toxicity of urea produced by *Ureaplasma* spp. (29). All metabolic diversity of *Mollicutes* contributes to the colonization of different niches and hosts.

The genome size also permits classification of the mycoplasmas into two groups. One is the genera *Mycoplasma* and *Ureaplasma*, with about 580–1,350 kilobase pairs (Kbp), and the others are the genera *Acholeaplasma, Spiroplasma*, and *Anaeroplasma* with 790–2,200 Kbp (14, 30–32). The non-essential genes were lost through genomic reduction. These microorganisms have evolved from Gram-positive bacteria by degenerative evolution to lose their peptidoglycan cell wall and some metabolic genes (33). Therefore, *Mollicutes* require additional supplements for laboratory growth. Thus, the uptake of extracellular nutrients including many biosynthetic precursors such as amino acids, nucleotides, and sterols are essential (34, 35).

In humans, *Mollicutes* usually cause urogenital and respiratory tract infections (36, 37). In animals, these bacteria prefer to colonize the mucous surfaces of the respiratory, genital, oropharyngeal, ophthalmic tract, as well as the mammary gland, and joints. They usually cause subclinical symptoms or can persist in healthy carrier animals maintaining a persistent parasitic lifestyle (20, 38). Pathogenic infections rarely lead to death; however, in some cases, the infection may be associated with chronic conditions due to the immunomodulation process in the affected region (18, 39, 40). The *Mollicute* cell membrane has a range of protein structures (Figure 1A), including lipid-associated membrane proteins (LAMPs) that represent important virulence factors. These structures will be described later in section: LAMPs and other surface molecules (41–43).

*Mollicutes* that infect herds are related to losses in farming, mainly disrupting animal weight gain and reproduction of cows, goats, pigs, chickens, and other species of agronomic interest (44–46). *U. diversum* is an important mollicute isolated from animals, and it induces a strong inflammatory response in the bovine respiratory and genital tract and is related to reproductive disorders. *U. diversum* has particular characteristics that make it challenging to control in infectious diseases in beef and dairy cattle, in addition to being a serious obstacle to artificial insemination and embryo transfer (47, 48).

## Ureaplasma Diversum

Bovine ureaplasmas (phylum *Tenericutes*, class *Mollicutes*, order *Mycoplasmatales*, family *Mycoplasmataceae* genus *Ureaplasma*) were first isolated in 1967 from tissue specimens of the vagina, urethra, and bladder wall of cows (49). For some time, these microorganisms were called *Bovine tiny (T)-strain mycoplasma*. This denomination reflected the morphological similarities with *U. urealyticum* cultures, at the time called *human T-strain mycoplasma* due to photomicrographs of agar *T* colonies published in the first report on these bacteria (50). In 1956, the term *T-strain* was applied to distinguish these microbes growing in solid medium with different morphology from other mycoplasmas described at the time (51).

In an attempt to establish distinguishing features, several studies were published comparing morphological characteristics and protein band profiles, by electrophoresis, from T-strains isolated from cattle with human isolates (52, 53). Serological tests showed differences between strains; however, the differences were not sufficiently marked to establish differences between species (53, 54). The protein profiles of ureaplasma by polyacrylamide gel electrophoresis showed non-shared peptides, indicating that they were different microorganisms (54). The confirmation was detailed in the DNA composition and the guanine plus cytosine (G + C) content. The human T-strain presented C+G 27.4 mol% (55) and bovine T-strains had 29.3% (52). Other T-strains were isolated from different animals including sheep and goats and the molecular methods allowed for these to be differentiated from bovine isolates (53, 56).

In 1974, the perception that isolated T-strains differed from conventional mycoplasmas prompted the creation of the genus *Ureaplasma* to encompass *Mollicutes* possessing the enzyme urease (51). “*Bovine T-strains Mycoplasma*” formed the species *U. diversum*. The species-specific epithet (*diversum*, from Latin meaning different) refers to the differences in polypeptides and G + C content compared to *U. urealyticum*, and the differences in the peptide profiles of the antigenic structures (determined by electrophoresis) from different isolates (54).

Many studies investigated serological grouping of *U. diversum* isolates. Initial studies using rabbit antisera, conducted in different laboratories by Howard et al. (57) and Ogata et al. (58), selected eight and seven strains, respectively, to represent the totality of antigens synthesized by ureaplasmas. Later, the new isolates, allowed for identifying expanding serotypes to eleven (59). Rabbit-produced antisera showed three distinct groups, two of which were serologically similar but not identical (57, 58). An important step in serological clustering was taken by Howard and Gourlay in 1981 when they selected one strain from each of the three antigenic clusters for antiserum production in calves (60). The antisera produced were tested in various *U. diversum* strains from different countries (Denmark, the United States, Canada, Ireland, England, and Japan), from symptomatic or healthy animals, and isolated ureaplasma from different anatomical regions (lung, foreskin, semen, urogenital tract, endometrium, and eye). The strains reacted with at least one of the three bovine antisera and were, therefore, classified into three clusters: cluster A, which is represented by strain A417; cluster B, represented by strain D48 or C, represented by strain T44 (59, 60).

Serotype distribution studies found that most strains were type-B strains isolated from cows with vulvovaginitis and infertility. However, there is no direct relationship between serotype and a specific disease (61). In general, all three serotypes are found in the reproductive tract of cattle (62). Despite the possibility of allocating isolates in clusters A, B, or C, Howard (63) previously warned that the serological relationship of antigens possessed by bovine ureaplasma determined by antisera or protein profile electrophoresis detect the relationship of some selected strains and not the complete dissemination of antigens present in *U. diversum*. In fact, studies by Marques et al. (64) reported high variability in the sequence of 16S rRNA gene fragments from ureaplasma isolated from healthy and unhealthy cattle. The significant number of single nucleotide polymorphisms (SNPs) found compared to the *U. diversum* sequence deposited at GenBank indicated intraspecific variability within these organisms. This variability may be translated into antigenic diversity of different strains and, consequently, generates different forms of interaction with the host (42, 64–66).

In morphological terms, *U. diversum* share the general characteristics of the *Mollicute* Class. They are pleomorphic (coccoid or coccobacillary), approximately ranging from 400 to 500 nm in diameter, microaerophilic (28, 54), with an optimal growth temperature of 37°C, but can grow between 33 and 37°C. The ideal growth pH ranges from 6.0 to 7.0. In a solid medium, they produce colonies of relatively small size like other microorganisms classified in the genus *Ureaplasma* (54). Observations from photomicrographs indicate that these colonies can range from 100 to 175 μm in diameter (67). Urea is an absolute requirement for growth. Hydrolysis of urea leads to ammonia formation; therefore, it increases *in vitro* pH (68), which is a striking feature of ureaplasmas differentiating them from other *Mollicutes* (2, 69). Although they also do not have a cell wall, they have a polysaccharide coating on the membrane forming a capsule 11–17 nm in diameter consisting of arabinose, xylose, mannose, galactose and glucose (28).

### Host Interaction With the Microorganism and Pathogenicity

*U. diversum* colonizes cattle mainly by the respiratory and genital/reproductive tract (47, 70–74). This microorganism is considered an opportunistic pathogen found in mucous membranes and secretions of the vulva, vagina, and udder of cows, causing severe granular vulvovaginitis, salpingitis, endometritis, mastitis, placentitis, and fetal alveolitis that may result in miscarriage or birth of weak calves (10, 31, 68, 75–77). The risk of infections is higher in younger cows, resulting in fewer births (78). Nevertheless, the infection is not dependent upon the presence of clinical symptoms, since the first isolates were, apparently, in the genital/reproductive tract of healthy cows (49). *U. diversum* was also isolated from the nasal cavity of asymptomatic calves (68). The presence of these microorganisms in healthy cattle herds makes it difficult to identify risk factors for infection, which makes establishing infection control strategies more difficult.

*U. diversum* can be sexually transmitted, and there is significant potential for horizontal and, in some cases, vertical transmission between animals (62, 79). One of the main forms of transmission is through coitus (natural reproduction), but infected bulls also spread ureaplasma through artificial insemination and breeding. The *Mycoplasma* spp. and *Ureaplasma* spp. are present in secretions, especially semen, preputial and vaginal mucus, conjunctival secretion, and milk (80, 81). Artificial insemination and embryo transfer are also potential sources of contamination (47, 80).

#### Pathogenicity in Cows

Granular vulvitis is common in ureaplasma-infected cows (82–85). The disease appears 1–3 days after vulvar inoculation in heifers (82, 83). Initially, acute infection involves mucopurulent discharge and mild vulvar epithelium hyperemia. The granularity can be classified as mild or severe. The mild granularity, usually starting from 24 to 48 h, appears laterally to the clitoris, with granules smaller than 1 mm in diameter and with light coloring. They are usually best observed with an oblique light source. Severe granularity is readily apparent without the need for additional light and usually involves the lateral vulvar wall as well as the clitoris area. The granules are usually red and larger, 1–2 mm in diameter (82, 83, 85). Heifers with severe granularity also have slightly edematous vulvar lips and purulent or mucoid discharge. Ureaplasmas are easily recovered from vulvar cultures of infected cows, fetuses, and calves after autopsy (47). Histological slides are characterized by focal and/or diffuse lymphocyte infiltrates both inside and just below the vulva mucosa, thus confirming vulvitis (47, 82, 83). In the vagina, vulva, and clitoris there are usually very serious lesions characteristic of Granular Vulvovaginitis Syndrome (GVS) (47, 85, 86).

During infection, the Fallopian tubes, uterus, cervix, vagina, and vulva fragments, as well as the lower reproductive tract, are slightly disturbed and present acute inflammatory process (82, 83). The histopathological findings of the reproductive tract of naturally infected *U. diversum* females are consistent with lesions that hinder fertilization (47, 87). Therefore, animals with GVS should be identified initially in the herds because, in addition to reduced fertility rates caused by tissue damage, they may contribute to spreading the microorganism (85). Chronic infection is also possible and can cause a variety of harm to animals (82, 83). Asymptomatic cows may chronically carry *U. diversum* and function as pathogen reservoirs and are, therefore, a major source of infection. Bovine ureaplasmas have surface compounds, such as variable surface lipoproteins (VSPs) that allow them to evade the host's immune system, invade cells, and help develop chronic infection (28, 42, 88–90).

Chronic GVS due to *U. diversum* if not diagnosed, may progress to endometritis (ascending route) and results in miscarriage or infertility (81, 91, 92). The descending pathway was also demonstrated by intrauterine and cervical inoculation in heifers. Inoculating these ureaplasmal strains in the uterus induces granulous vulvitis, endometritis, and salpingitis. Cervicitis can occur between 2 and 5 days after inoculation. Cervical damage alters the pH of the local mucus and provides an adverse environment for sperm survival (82, 83). Thus, the pathogenesis in the reproductive tract of cows should be considered significant, as granular vulvitis, endometritis, salpingitis, or cervicitis may result in reproductive losses (92). In oviduct tissue specimens, the most frequent lesions observed histologically are epithelial desquamation with basement membrane exposure, lumen clot formation, curvature, or absence of cilia, as well as a cytoplasmic projection of many epithelial cells toward the lumen (82, 83). In the cervix and uterine body, the lesions are also severe, destroying the epithelial cell cilia and resulting in ulcerated areas. Epithelial lesions such as vacuolization of epithelial cells, spongiosis, loss of epithelium with basement membrane exposure, and lymphocyte accumulation are observed in fornix tissue specimens histologically (85, 92).

Because ureaplasmas have no rigid cell wall and require a microaerophilic environment to grow, the survival of these organisms is compromised in the womb (93). However, more favorable situations, such as in the estrous cycle, when the cervix is open, or during artificial insemination, the infection becomes more probable. *U. diversum* seeks whole cells for parasitism since adherence and parasitism of epithelial cells are essential conditions for their survival. Supporting these findings Lingwood et al. (94) showed that *U. diversum* is able to bind to the surface of male sperm and in endometrial cells. Later Kim et al. (95) established an *in vitro* model of endometrial infection. These early studies supported the growth of ureaplasmas within uterine endoemetric cells. The local effect of infection is likely to occur through the process of constant reinfection rather than the release of other opportunistic organisms (85). In humans *Ureaplasma* spp. were also detected by next-generation sequencing (in a study that also used PCR and culture for detection) as the most prevalent uterine colonizer in pregnant women with and without chorioamnionitis, specifically, individuals in premature births with severe chorioamnionitis had high abundance of *U. parvum* (96). There is also a positive association between the detection of *U. parvum* in samples of placental tissue and abortion (97). Therefore, these studies suggest that ureaplasmas that infects humans or animals can colonize/infect the placenta and amniotic fluid for long periods during pregnancy in the absence or presence of adverse outcomes.

*U. diversum* infection/colonization in fetal lungs or after the endobronchial inoculation of this ureaplasma is associated with abortion and newborn death in cows. The death of calves may be linked to the development of catarrhal lobular pneumonia (98–100). A high prevalence of *U. diversum* has been detected in cases of abortion, calf morbidity (Table 1). Pulmonary pathology in fetuses usually presents with lymphocyte infiltration and conjunctivitis (92, 100, 104, 106, 107, 111, 112). Also, typical features of an abortion caused by *U. diversum* include a relatively fresh fetus and frequently retained and inflamed placenta (113). Postmortem examinations performed on fetuses, late abortion, and weak-born calves tested positive for *U. diversum* in tissue specimens collected from the placenta, lungs and abomasal fetal fluid. These findings were accompanied by diagnoses of placentitis, conjunctivitis, and pulmonary pathology, suggesting the establishment of an immune-mediated response. In these animals, the presence of neutrophilic bronchopneumonia may range from moderate to severe and may be diffuse or acute (47).

**Table 1:** *U. diversum* prevalence in countries, animal health, anatomical site, and detection method.

**Country**	**Animal**	**Animal Status**	**Specimen**	**Prevalence (%)**	**Method**	**References**
Argentina	Bulls	Healthy	Penis Foreskin	64.7	PCR	(74)
Australia	Cows and Bulls	Asymptomatic or with a genital lesion	Penile mucosa swabs Swabs of the clitoral fossa and vaginal mucosa	35.2	PCR	(101)
Australia	Cows and Bulls	Asymptomatic or with a genital lesion	Penile mucosa swabs Swabs of the clitoral fossa and vaginal mucosa	15	Culture /PCR	(70)
Australia	Bulls	Asymptomatic or with penile and preputial lesions	Semen	31.03	Culture /PCR	(102)
Austria	Cows	Vaginitis	Cervical or vaginal swab	35.5	Culture	(77)
Brazil	Cows	With vulvovaginitis or healthy	Vulvovaginal swab	46.42	qPCR	(68)
Brazil	Cows	With vulvovaginitis or healthy	Vaginal Mucus Swabs	41.1	Culture	(103)
Brazil	Cows and heifer	With granular vulvitis	Vulvovaginal Swabs	38.8	Culture	(10)
Brazil	Calves	Sick or healthy	Respiratory tract	20	PCR	(104)
Brazil	Cows	Vulvovaginal Injuries	Vaginal Mucosa	64.0	PCR	(87)
Brazil	Cows	Asymptomatic	Vaginal Mucus Swabs	18	PCR	(87)
Brazil	Cows and heifer	With vulvovaginitis	Vaginal swabs	83.9	Nested-PCR	(105)
Belgium	Calves	Recurrent respiratory disease or other diseases	Lungs sample	14	Culture	(106)
Canada	Calves	Dead or euthanized for the first 60 days	Necropsies: lung tissue	25	Culture	(107)
Canada	Swine	With mycoplasma-type cough	Lung, trachea and other tissues	3.85	qPCR	(108)
Cuba	Swine	Pneumonic	Pneumonic lung samples	6.6	PCR	(72)
Finland	Calves	Miscarriages, stillbirths or neonatal deaths	Necropsy	13	Culture	(109)
Poland	Calves	Stillbirths	Lung	0	PCR	(110)

Milk production is also affected mainly due to the development of bovine mastitis, inflammation of the mammary gland, which is one of the main diseases in dairy herds (114). Bovine ureaplasma produces clinical mastitis along with visible changes in milk and udders (115). Mastitis caused by *Mollicutes* is less common than mastitis caused by other bacteria, but results in severe udder disease (116), in dairy herds, *Mollicutes* can cause clinical, subclinical or chronic mastitis (117). When experimentally inoculated in the udder of cows *U. diversum* excretion and an increase in the number of cells in milk were detected. In some cases, milk secretion ceases completely. Histopathological examination of the udder sections reveals neutrophil infiltration along with interstitial hyperemia (115), This is a major problem for milk production and animal welfare in large dairy herds (114).

#### Pathogenicity in Bulls

In bulls, *U. diversum* is involved in cases of seminal vesiculitis, balanoposthitis, epididymitis and other pathologies caused by morphological and functional changes in sperm (88, 91, 102). Ureaplasmas can colonize both the proximal and distal portions of the urethra and the preputial cavity, which allows for semen contamination (102, 118). The preputial cavity and urethra appear to be the main means of semen contamination. Thus, procedures to reduce the number of microorganisms in the foreskin may not be effective enough in preventing semen contamination (119). Several studies have identified *U. diversum* in bull reproductive tract fluids including fresh semen (120, 121), preputial mucus samples and distal urethral secretion (74). Microorganism adherence interferes with spermatogenesis, sperm transport, capacitation, and fertilization (102).

Bulls positive for *U. diversum* but without genital lesions may have abnormally-tailed (folded and coiled) sperm, as well as sperm surface abnormalities (small bulges). They also have irregularities and infection of bacteria in the entire length of their bodies from head to tail. This suggests impaired sperm function and possibly infertility (74, 102).

#### Interference With Artificial Insemination (AI) and Embryo Transfer

Some viral, bacterial, protozoan and parasitic organisms may be transmitted through bovine semen. In some cases, the presence of infectious agents is sufficient for their transmission to heifers or cows through natural reproduction or artificial insemination (AI) (122).

Le Grand et al. (121) demonstrated a high frequency (74%) of ureaplasma in the semen of bulls intended for AI. In this sense, *U. diversum* infects semen destined for AI, colonizes blastocysts and fetuses and induces gestational losses or birth of weak calves. In studies by Crane and Hughes, (47) the losses between suspected or confirmed embryogenic pregnancies for infection with this bacterium were 67%. In these studies heifers did not have fertility problems, rather pregnancy loss and stillbirths were the prominent clinical problems. This suggests that embryos are the source of the *U. diversum* infection, abortions, stillbirths, and in weak neonates could be how the disease manifests.

In AI, it is important to control the population of microorganisms in the semen and, thus, prevent the introduction of diseases in individual animals or herds (123). However, foreskin in healthy semen donors contains numerous bacterial species that can mix with semen during ejaculation and during collection. Some of these bacteria can pose a significant risk for inseminated females. Therefore, it is important that control is achieved through the use of antibiotics and appropriate hygiene techniques (124). Along with the advent of AI techniques, embryo processing techniques have been developed to reduce the risk of transfer of infectious diseases by bovine embryos derived from this technique (125). Ureaplasmas are recognized as one of the few potential pathogens that are not removed from embryos through the IETS (International Embryo Transfer Society) recommended washing and cleaning procedures (113, 126).

Penicillin and streptomycin as well as gentamicin are the most common antibiotics used to control microorganisms in embryo transfer techniques, without any apparent effect on the development of embryos of domestic animals. They are effective against a variety of Gram-positive and Gram negative bacteria. Generally these antibiotics are used in combination with each other (124, 127). Despite this, for structural (as already discussed) and resistance issues, standard antibiotics present in embryo processing media are not effective against *Mollicutes* (124). Therefore, antibiotics that act on the cell wall, such as penicillins, do not act agaisnt *U. diversum* (due to the absence of a cell wal in these bacteria). In theory, antibiotics that are unable to penetrate biological membranes (such as gentamicin) would also not be 100% effective against bovine ureaplasmas because these microorganisms are capable of embedding themselves in sperm and blastocysts (42, 102). Some alternatives to fight mycoplasmas are being developed, such as an enzymatic treatment (128), immunological methods (with the use of specific antibodies against the infectious agent) and photosensitive dyes and chemical compounds with germicidal effects (124, 127, 129). All of these alternatives can contribute to the development of effective control methods against molicutes that infect semen and embryos destined for AI and embryo transfer.

Forms of contamination during the embryo transfer procedure are also possible. *U. diversum* present in the vulva of donor cows (cows from which the embryos will be removed) can contaminate the catheter or discharge solutions and remain in recovered bovine embryos. *U. diversum* can also be carried in the vulva of donor cows or recipient cows (cows that will have an embryo inserted in their uterus) to the animal's uterus during AI and embryo transfer (47). Veterinary diagnostic laboratories generally do not use polymerase chain reaction (PCR) methodologies to detect *Ureaplasma* spp.; most of the time the culture method is used. Therefore, some abortions caused by *U. diversum* can be misdiagnosed. Colonization of the reproductive system at the time of embryo collection allows *U. diversum* to adhere and disturb embryo development (47).

Gaeti et al. (87) found by PCR methodology a conception rate for embryo transfer of 64.8% in heifers positive for ureaplasma, including those with vulvar lesions. This prevalence is similar to the average conception rates of embryo transfer programs in cattle ranging from 50 to 60.4% (130, 131). Indeed, these percentages suggest initially that *U. diversum* does not affect conception rates and that embryo transfer does not appear to increase the risk of infertility among infected heifers (87). However, a good conception rate may not correlate with the absence of miscarriages and the birth of healthy calves. In humans the infection by *Ureaplasma* species was associated with chorioamnionitis (regardless of gestational age), spontaneous abortions or miscarriages, and neonatal respiratory diseases. The presence of these microorganisms in the upper genital tract of non-pregnant women suggests that these microorganisms may infect the embryo at the time of implantation and can adversely affect the health of the pregnancy and neonate (132–134). *U. diversum* may remain in cells internalized at early fetal development, such as in the blastocyst, without cytopathic effects (42). Thus, placenta, ocular conjunctiva, and lung tissue could be colonized at late stages of development or after calving; thus causing pulmonary placentitis, conjunctivitis, and pathology in calves (47).

#### Emerging Pig Isolation Cases

*Mollicutes* usually exhibit high specificity to host tissues. However, some *Mycoplasma* ssp. have been isolated from unusual hosts (18, 135, 136). It is possible that by overcoming the species barrier, *Mollicutes* have colonized other hosts and become pathogenic regardless of their phylogenetic distance (135).

For many years now, *U. diversum* has been isolated from cattle and associated with various diseases in the genital and respiratory tract, but the ability of these bacteria to colonize swine has recently aroused interest in the scientific community. In 2013, Lobo et al. (71) isolated *Ureaplasma* spp. of pig lung with typical lesions of enzootic pneumonia. The association of ureaplasma with porcine lung lesions led to studying *U. diversum* in these hosts. In 2014, the first study reported that *U. diversum* may infect swine and be associated with pneumonia. However, unlike cattle, these bacteria were only isolated from sick animals (72).

In swine, *U. diversum* showed a higher affinity to lung tissue (72, 108). In Canada, a study in tissues/organs of swine detected *U. diversum* only in lung tissue. In the same study a coinfection with other mycoplasma and ureaplasma species such as *M. hyopneumoniae, M. hyorhinis* was also identified (108). The role of *U. diversum* in swine remains unknown. The main techniques used to identify *U. diversum* in pigs have been conventional PCR or qPCR. Although these techniques have high sensitivity and specificity, the inclusion of serological tests can contribute to a better characterization and knowledge of the swine isolates. Therefore, it is not yet possible to determine whether this species is an opportunistic pathogen or a primary agent involved in Swine Respiratory Complex diseases (71, 72, 108).

### Impacts on the Livestock

Reproductive disorders, animal development and positive cultures to *U. diversum* or association with other bacteria and viruses, makes this ureaplasma an important threat to raising cattle for large producers (86, 118). This requires special care and quality control for meat production and dairy activity in countries such as Brazil, the United States, China, and India (137–141).

Artificial insemination, embryo transfer, *in vitro* embryo production and the use of somatic cell nuclear transfer or cloning has delivered several benefits to herd productivity (142). However, these techniques do not eliminate the occurrence of *Mollicutes* and reproductive disorders in cattle (143, 144).

Several *Mollicutes* have been isolated in the bovine urogenital and respiratory tract or aborted fetuses, including *M. bovis, M. bovigenitalium, U. diversum*, among others (11, 93, 104, 110, 114, 145). *U. diversum* is related to reproductive disorders, weight loss and reduced milk yield in cattle. These disorders also increase veterinarian and pharmaceutical costs causing significant economic losses in the livestock sector (79, 146, 147).

### Diagnosis

The first techniques used for *U. diversum* diagnosis were culturing and producing typical colonies, urea hydrolysis, specific serology, and DNA or protein profiles. The medium, known as Hayflick's medium, presently used in most diagnostic labs for *U. diversum* identification has undergone few changes over the years. Hayflick's medium contains a combination of mycoplasma broth, yeast extract, horse serum, urea crystals, phenyl red, and penicillin (42, 59). The urease activity and ammonia production raises the pH of specific broths. In solid medium the precipitation of cations Mn, due to urease activity, produces brown-colored colonies (10, 148). Usually with lysed isolates and the immunodiffusion methodology, ureaplasma are serologically characterized with polyclonal antisera, usually rabbit or calves (79). Some studies have shown that molecular diagnosis methods provide better results to detect bovine ureaplasma. PCR assays have shown high specificity and several recently published studies have used the rRNA 16S gene as a basis for primer construction (68, 79, 149).

Conventional PCR using primers UD1 (forward: 5′-CCG GAT AAT AAC ATT TTG-3′) and UD2 (reverse: 5′-CCT TGC GGT AGC ATC GA-3′) as well as nested PCR using UD3 (5′-AAT GTC GGC TCG CTT ATG AG-3′) and UD4 (5′ -CCT GTC ATA TTG TTA ACC TCC GC-3′) were tested to identify *U. diversum* in animals with signs and symptoms of granular vulvitis or reproductive dysfunction. Nested PCR diagnosis showed high specificity (73.1%), efficiency (82.7%), and sensitivity (100.0%) compared with culture method: metabolites and serological tests (79). This PCR methodology, with use of the same pairs of primers targeting the gene encoding 16S rRNA, was used by Buzinhani et al. (86) for detecting *U. diversum* in isolates from different Brazilian farms and also showed a higher sensitivity compared to culture techniques.

Marques et al. (68) developed primers and TaqMan probes based on the 16S rRNA gene for quantitative detection of *U. diversum* by real-time PCR (qPCR). The test that was developed demonstrated high sensitivity and specificity for ureaplasmal detection and quantification in swab samples, being able to detect quantities as low as only 10 copies of the genome/reaction. The qPCR methodology was 100 times more sensitive than conventional PCR, and there was no cross-reactivity with other *Mollicutes* or eubacteria. In fact, the qPCR sensitivity and specificity make this technique useful for the diagnosis of *U. diversum* as also mentioned in other studies, as shown in Table 1 (108, 149).

*Mollicutes* in general are fastidious bacteria and difficult to culture and detect; this hinders the collection of epidemiological data and understanding of pathogenesis (10). The importance of *U. diversum* infections in cattle, the use of PCR in diagnosis, and sharp detection of this mollicute in clinical materials will help better control this microorganism (10, 68, 79, 86, 149).

### Prevalence

The prevalence varies according to several factors (Table 1) including several important ones that follow: (1) the cattle population size in a country studied. In Brazil, which has one of the largest cattle herds in the world (150), prevalence may even vary between different regions; (2) health status of the host animal (healthy or unhealthy); (3) sex and stage of animal development (cow, bull, young calf, calf, fetus); (4) anatomical area studied (regions of the lower or upper reproductive tract, respiratory tract, eye conjunctiva) and (5) the diagnostic technique used (culture, PCR). The variations and prevalence of *U. diversum* are presented in Table 1, which compiles some data on the recent infection prevalence in cattle and swine.

## Virulence of *U. DIVERSUM*

*Mollicutes* have virulence mechanisms and also use host cell nutrients. Virulence factors include the following: (1) the production of toxic primary metabolic compounds such as ammonia or oxygen hydroxide (2) Adhesion and invasion in host cells; (3) LAMP compounds, and (4) modulation of apoptosis mechanisms. Many of these mechanisms have been studied in *U. diversum*; however, some virulence and pathogenic factors remain unexplored (28, 42).

### Urea Production and Modulation in Prostaglandin Synthesis

Ureaplasma do not have a complete arginine dehydrolase pathway (151). Thus, urea hydrolysis plays an important role in energy metabolism of ureaplasmas and is an important energy source through the production of ATP (152–154) as shown in Figure 2. Ajello et al. (155) showed that ammonia can be produced by the degrading L-histidine. This substance can also be produced by the activity of L-histidine ammonialyase detected in ureaplasmas. There is no data in the literature on the pathogenic effects of the expression of the urease gene by *U. diversum*; however, in these bacteria, the urease genomic cluster was identified, with more than 90% homology to the human *Ureaplasma* spp. coding sequence (CDS) (28, 90). Hydrolysis of urea in ureaplasma generates an electrochemical gradient through accumulation of intracellular ammonia/ammonium. The gradient fosters a chemiosmotic potential that generates ATP (156). Thus ATP production is associated with three enzymatic components: urease, (three-subunit urease+accessory proteins), an ammonia/ammonium transporter, and a FOF1-ATPase– Figure 2—(28, 153, 154).

Ureaplasmal ammonia release in a tissue irritates the mucous membranes of the urogenital and respiratory tracts. The mucosal cells also have targets for adhesion and colonization of ureaplasma strains (153, 154, 157, 158). In addition, the ammonia may also intoxicate adjacent tissues.

Another virulence factor is the significant decrease in prostaglandin E2 and F2a by endometrial cells after ureaplasmal infection (95). Prostaglandins are necessary for implantation of embryos and maintenance of pregnancy in cattle (95, 159). Ureaplasmas interfere with prostaglandin biosynthesis due to their phospholipases in membranes (160). The genes for phospholipase D family proteins and triacylglycerol lipases in bovine ureaplasmas may increase the release of arachidonic acid and inhibit the substrate for prostaglandin synthesis (28). The phospholipase D gene was found in 40% of 45 *U. diversum* isolates from different regions of Brazil (65). The presence of prostaglandins in humans and other animals has also been associated with other *Mollicutes*. These molecules were observed in human fetal membranes infected by *U. parvum* (161).

Prostaglandins are strong regulators of cellular activities in reproductive processes. Prostaglandins are necessary for implantation of embryos and maintenance of pregnancy (160). Alteration of this context by ureaplasmas, probably by the activity of phospholipases, can induce labor (153). Prostaglandins can have a pro-inflammatory action on the host's reproductive system; inflammation ultimately initiates preterm labor (162). This is in line with several studies associating bovine *U. diversum* with premature births (47, 86). Despite this, the role of phospholipases in *U. diversum* needs to be studied in more detail.

### LAMPs and Other Surface Molecules

Structurally the cell membrane of *Ureaplasma* spp. has the following three layers: two border electron-dense layers with a middle less dense layer, which interacts with the external environment, as shown in Figure 1 (163, 164). LAMPs represent a mixture of cell surface-expressed mycoplasmal lipoproteins interacting with the host cells and are the major molecular patterns associated with pathogens in various mollicute species (16, 164, 165). They are attached to the membrane mainly by electrostatic interactions and thus can be easily released (16, 166). A large number of expressed lipoproteins account for a significant portion of the membrane mass (16, 167–169). These LAMPs play an important role at the site of infection causing inflammation by activating or inhibiting apoptosis: Probably through the breakdown of extracellular ATP into adenosine, this inhibits the growth of various types of cells and even induces apoptosis (170, 171); in the ATP-binding cassette (ABC) transport system (172); in the virulence of different strains, mainly due to the fact that many LAMPs act as VSPs (173, 174), in cell adhesion, as described in section Cell adhesion and invasion (175, 176), and in immunomodulation of the host response, as described in the section Inflammation and Immunomodulation (42).

In the bovine ureaplasma genome, genes encoding 37 unknown lipoproteins were found, as well as one VSP with 36% identity with the *M. pulmonis* variable surface antigen lipoproteins (VsA) (28, 90). VSPs undergo constant variations (16, 177). A variety of mechanisms may be involved in these antigenic variations, including phase variation (178), variation in the number of tandem repeats at the carboxylic terminus (179, 180), masking by another surface antigen (181), and (177). Studies with other *Mollicutes* show that VSPs are related to cell adhesion (182, 183), and induce proinflammatory or anti-inflammatory response (175).

The presence of multiple band antigen (MBA) coding genes in *U. diversum* plays a role in antigenic variation as mechanisms for rapid adaptation to changes in the microenvironment (28). The MBA contains a signal peptide and an acylation site in the N-terminus. The C-terminus is composed of multiple serial repeats (184, 185). Uchida et al. (186) demonstrated that MBA from *U. parvum* is a potential virulence factor and causes intrauterine fetal death and preterm delivery after injection into the uterus on day 15 of gestation in pregnant mice. This study also showed that the N-terminus diacylated lipopeptide is essential for the onset of inflammation that causes reproductive disorders (186). Antigenic variation of MBA has not yet been investigated in *U. diversum*, but in *U. parvum* these variations are correlated with the severity of the infection. Knox et al. (187), using a pregnant sheep model, showed that larger numbers of *mba*/MBA size variants generate low inflammation response within the chorioamnion and little or no chorioamnionitis. In ureaplasma isolated from human placentas, the variation in the size of *mba*/MBA was associated with a reduced incidence of histological chorioamnionitis and significantly lower levels of the cord blood proinflammatory cytokines (134). In this way, by avoiding the immune recognition of the host through antigenic variations, so that ureaplasmas collaborate for a less severe infectious pattern that would cause choriomyionitis, and on the other hand, these microorganisms are established as an asymptomatic chronic infection (188). In the intrauterine fetus, this establishment may cause chorioamnionitis, preterm birth or fetal death (134, 186, 188).

In the *U. diversum* genome, genes encoding hemolysin were found, being an enzyme that causes blood cell lysis, and MIB-MIP system genes (MIB: Mycoplasma Ig binding protein; MIP: Mycoplasma Ig protease). MIB acts as an IgG-binding protein, while MIP cleaves the IgG heavy chain (28, 90). *U. diversum* genome has several CDS for potentially immunogenic molecules (LAMPs, VSPs, MBA, hemolysin, and MIB-MIP system); however, the role of most of these molecules has not yet been thoroughly investigated. Identifying the function of these immunogenic molecules may represent a significant advance in the understanding of the pathogenesis caused by ureaplasmas. Modification of some membrane antigens can provide an effective strategy to evade the host immune system (189).

### Cell Adhesion and Invasion

*U. diversum* is a facultative intracellular microorganism (88). The adhesins are responsible for adhesion and facilitate the process of cell invasion. These molecules also enable the ureaplasmas to use the host cell nutrients and facilitate the cell invasion process. Invasion is a complex process involving mollicute adhesins and cell receptors (190). Once adhered, these bacteria interact with membrane receptors or interfere with the transport mechanism of host cells rendering them vulnerable to cytotoxic metabolites and cytolytic enzyme activities (190, 191).

Macromolecules of different *Mollicutes* have been identified and related to cell adhesion. Among the most commonly studied are *M. pneumonia* P1 and P30 proteins (23, 192), and *M. gallisepticum* GapA and CrmA proteins: cytadherence-related molecules (193). In *M. bovis* proteins P26 (194), α-enolase, adhesion-related factor (195), and members of the VSPs family with adhesion function were identified (175). The CDS for various surface proteins have been found in the *U. diversum* genome. In addition, the presence of capsules and LAMPs were associated with increased virulence and promote adhesion to the host cell (28).

The invasiveness is closely related to cell adhesion (31, 36). The invasion has already been described for several species of *Mollicutes* including cellular invasion by M. *penetrans, M. genitalium* (196) and, *M. gallisepticum* (197). Cellular internalization by *U. diversum* was first described by Marques et al. (198) using Confocal Laser Scanning Microscopy. Internalization in Hep-2 cells was detected 1 min after infection. The microorganisms after 3, 8, and 24 h were detected around the perinuclear regions; however, the nuclear invasion was not verified (198). The invasion in the bovine sperm described by Buzinhani et al. (88) emphasizes the role of these microorganisms in reducing sperm motility and in causing seminal vesiculitis, and epididymitis. Regarding the reduction of reproductive efficiency, Santos-Junior et al. (42) demonstrated that *U. diversum* is able to embed within blastocysts and induce higher gene expression of interleukin 1 beta (IL-1β) and tumor necrosis factor alpha (TNF-α) compared to uninfected blastocysts. This suggests the existence of an early signaling system to respond to bacterial infections capable of protecting embryos against mollicute infections (199).

Cell invasion is an important virulence factor in *Mollicutes* and can be considered a strategy for persistence in immunocompetent hosts. These hosts also provide essential nutrients for *Mollicutes* (89, 198, 200, 201). Intracellular localization also protects *Mollicutes* from antibiotics and hinders their elimination from the infected cell cultures (2). Gentamicin is an example of an antibiotic that cannot penetrate mammalian cells when in low concentrations, therefore, this antibiotic is not effective against *U. diversum* and other internalized *Mollicutes* in mammalian cells (198). The mechanisms inherent in the adhesion process have not been completely elucidated in *Mollicutes*. Some approaches show that plasminogen plays an important role in *M. fermentans* invasion. When *M. fermentans* is exposed to plasminogen, they are able to invade HeLa cells; however, the unexposed cells lose their invasive abilities, showing the plasminogen dependence on the invasion process (202). Phospholipases are also related to cell invasion; these enzymes, already identified in *U. diversum*, are able to cleave membrane phospholipids and gain access to the host cell cytoplasm (191, 198, 200, 203).

### Apoptosis

The LAMPs of *Mollicutes* may also modulate apoptosis mechanisms (171). *Mycoplasma bovis* induces apoptotic death of bovine lymphocytes through a mechanism in which lipoproteins play an important role (204). Several other *Mollicutes* demonstrate the ability to modulate apoptosis in different cell lines (205–207).

The ability of *U. diversum* to induce cellular apoptosis was found after inoculation in Hep-2 cells. The number of apoptotic cells was higher than uninfected cells. However, the number of apoptotic cells decreased over time. Such reduction may be related to the persistence of this microorganism in the intracellular environment where it can be protected from host immune factors (89). In infectious diseases, apoptosis can be characterized as a defense mechanism against intracellular microbes that do not necessarily have any mechanism to impede them. However, excessive apoptosis of immune cells can affect the immune response at the primary site of infection and thus facilitate the diffusion of mycoplasma (205, 208).

## Inflammation and Immunomodulation

The immune reaction against *Mollicutes* is mediated by T and B lymphocytes (209). Therefore, it is suggested that a chronic adaptive response develops with consequent immunoglobulin secretion, antibody opsonization, complement system activation and infiltration of neutrophils and macrophages (85, 210).

*U. diversum* in bovine vaginal lesions revealed epithelial necrosis, infiltration and cell proliferation (85). In uterine tissue specimens, the glandular epithelium is damaged by infiltrating leukocytes and eosinophils in the submucosa. Edema, connective tissue proliferation, muscle fiber necrosis, and small areas of epithelial ulceration may be observed histologically (82, 83). Pulmonary histopathology of fetuses and calves is characterized by significant infiltration and accumulation of lymphocytes throughout the lung, near the vessels and bronchioles, as well as in the alveolar tissue (47).

*Ureaplasma* spp. surface proteins are the main initiators of the inflammatory response during infection (28, 42, 164, 183). The interaction between LAMPs and toll-like receptors (TLRs) initiates signal transduction pathways that promote inflammation-related gene transcription (211). However, an anti-inflammatory profile can also be established. The ureaplasma strains may induce the production of different cytokine profiles depending on the surface antigens (41, 43).

### Proinflammatory Profile Induction

In several *Mollicutes* the production of proinflammatory cytokines is associated with interactions of LAMPs with TLRs (41). The signaling pattern differs among *Mollicutes* and the TLRs are part of the signaling process (41, 212, 213). Therefore, intracellular recruitment of myeloid differentiation factor 88 (MyD88), activation of nuclear factor-κB (NF-κB) and activator protein 1 (AP-1) occurs (41, 211). These transcription factors play a central role in the induction of cytokine and chemokine profiles (214, 215), such as interleukin (IL-8), Monocyte Chemoattractant Protein-1 (MCP-1), Macrophage-1a Inflammatory Protein (MIP-1a), Macrophage and Granulocyte Colony-Stimulating Factor (GM-SFs), as well as prostaglandin and nitric oxide (18, 216). These molecules are associated with a strong inflammatory response (217–219).

Inoculation of viable or inactivated *U. diversum* in bovine macrophages increases TLR gene expression and association with IL-1β and TNF-α production, and induction of a strong inflammatory state (28). These findings are consistent with the clinical status of cow infections. These cytokines appear to play a central role in the response to *Mollicutes*. Several studies have described their involvement both *in vivo* (220) and *in vitro* (41, 221, 222). Human mononuclear cells infected with *Mycoplasma pneumoniae, M. hyorhinis, M. arginine, M. salivariu*m, *M. orale, M. gallisepticum* also have increased expression of IL-1β and TNF-α (169).

Silva et al. (223) found that *U. diversum* intrauterine infection in a mouse model induced TNF-α production. *U. diversum* was also able to induce TNF-α, IL-1β, and IL-6 production in macrophage culture (224). Inoculation of viable or inactive strains and different concentrations of LAMPs induced higher expression of IL-1β, TNF-α, TLR2, and TLR4 gene in bovine macrophages (42). A common feature of these studies was the dose-dependent relationship (even to microbial load or LAMP amount). Thus, as suggested by Marques et al. (68), The higher bacterial load of *U. diversum* may be directly related to the intensity of the clinical conditions of infected cows.

In infections due to *Mollicutes*, the signal transduction leading to the production of proinflammatory cytokines indicated is dependent on NF-kB or AP-1 (225). Many studies show that stimulation of TLR1, TLR2, and TLR6 by LAMPs results in the activation of these transcription factors. TLR2 is known to form heterodimers with TLR1 (TLR2/TLR1) and TLR6 (TLR2/TLR6), thereby distinguishing different microbial components, including *Mollicutes* LAMPs (226–228). LAMPs derived from *M. genitalium* can activate NF-kB via TLR1, TLR2, and TLR6 (211). The same occurs in THP-1 cells infected with *M. pneumoniae* (229) and in macrophages infected with *U. parvum* LAMPs (164, 230). Wang et al. (41) found that overexpression of TLR2 and MyD88 in bovine embryo lung cells depends on the stimulation by *M. bovis* LAMPs and that these lipoproteins activate IL-1β production via NF-kB via TLR2 and MyD88.

**Figure 3:**
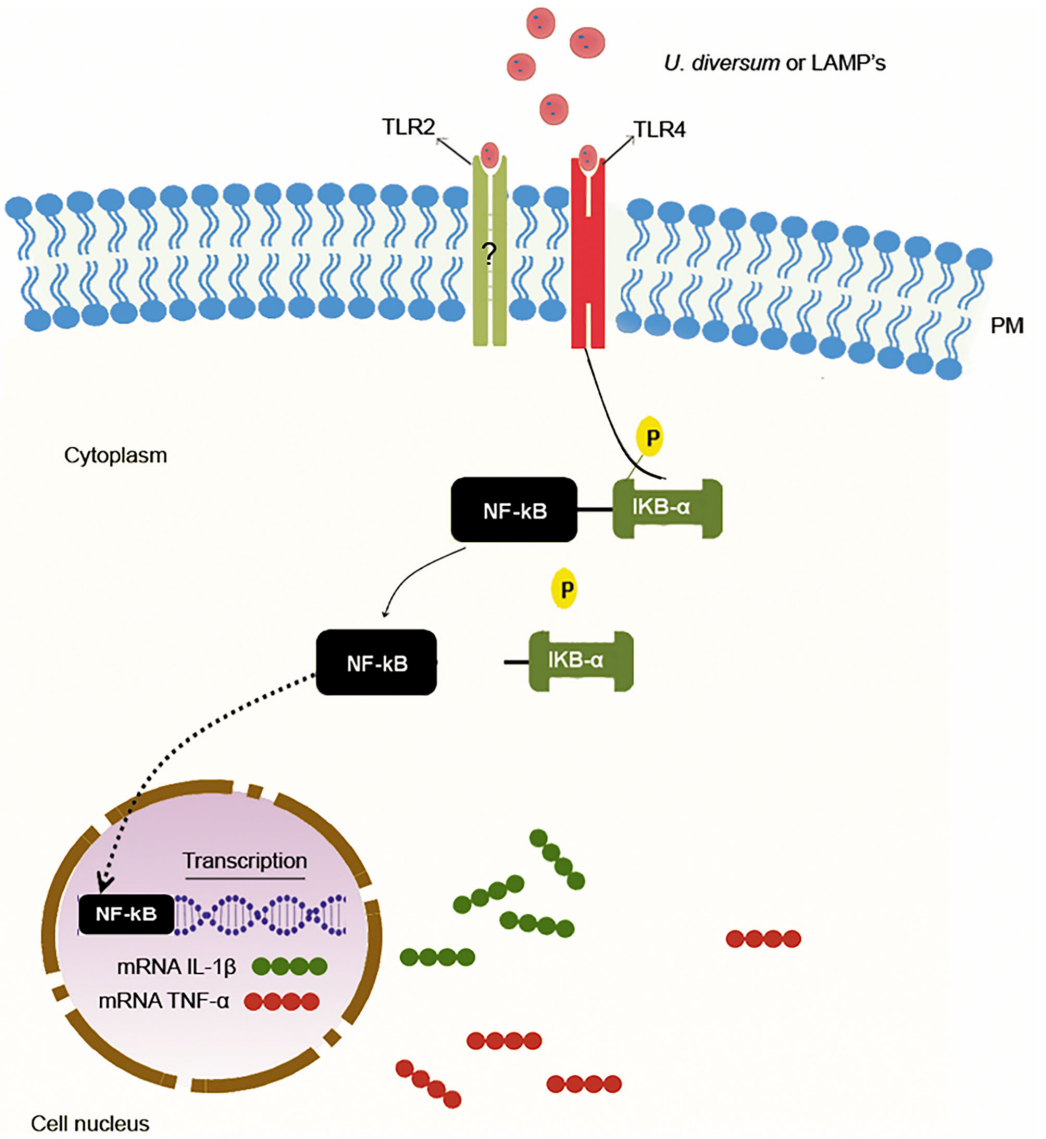
Proinflammatory response induction mechanism by *U. diversum*. The interaction of *U. diversum* or LAMPs activates NF-kB. Once activated, NF-kB translocates to the nucleus and acts as a transcription factor for IL-1β and TNF-α.

*U. diversum* also induces high TLR2 expression in murine and bovine macrophages (28, 42). This demonstrates the fundamental role of this receptor in signaling pathways that lead to the production of a proinflammatory cytokine profile. However, studies evaluating the expression of TLR 2 and TLR 4, as well as the use of TLR4, TLR2/4, and NFK-B blockers associated with TLR2 (Pam3CysSK4) and TLR4 (LPS) ligand, have shown that TLR4 has an important function in signaling that leads to the expression of IL-1β and TNF-α. In this case, the TLR4 blockade inhibited the expression of these cytokines, suggesting that *U. diversum* and its lipoproteins interact with TLR4 in signaling that acts via NF-kB to stimulate the inflammatory response, as shown in Figure 3 (42). However, due to the antigenic variety of *U. diversum*, it is possible to suggest that other signaling pathways may coexist.

Other studies have investigated TLR4 signaling in *Mollicutes*. Shimizu et al. (231) showed that *M. pneumoniae* induces strong inflammatory responses in TLR2 knockout mouse macrophages and the inhibited response in TLR4 inhibitor treated macrophages suggesting the establishment of TLR4 mediated inflammatory response. Both TLR2 and 4 may be involved in signaling by some *Mollicutes*. It has been found in *Mycoplasma arthritidis* that TLR 2 and TLR4 interact with HLA-DR by increasing the binding and antigen presentation of this mollicute to murine T cells (213). Furthermore, *U. urealyticum* LAMPs interact with TLR-2 and TLR-4 leading to immunomodulation with the release of proinflammatory mediators (212).

### Induction of an Anti-inflammatory Profile

LAMPs can also downregulate the immune system. *M. fermentans* lipoproteins have been shown to induce IL-10 production in human monocytes (232). Combining *M. flocculare* with *M. hyopneumoniae* also increases the ability of bone-marrow-derived dendritic cells (BM-DC) to secrete IL-10 (233). IL-10 may act as an anti-inflammatory cytokine necessary to maintain cellular homeostasis or favor the Th2 pathway by inhibiting IL-12 production (31, 234).

Andrade et al. (65) noted that some *U. diversum* strains induced significant expression of IL-10 and IL-17 when inoculated into bovine macrophages. The expression of IL-17 does not prevent anti-inflammatory modulation. Thus, the high antigen variability contributes to *U. diversum* modulating the immune system in different ways. This type of signaling is common in some *Mollicutes*; *M. bovis*-infected bovine monocytes, with increased IL-10 secretion (235).

Despite the role of IL-17 in neutrophil recruitment, it can also act synergistically with IL-10. Corroborating this theory Jimbo et al. (236) showed that IL-17 does not increase bovine neutrophil survival after *M. bovis* infection. Thus, the increase in IL-10 and IL-17, reported in *U. diversum* infected bovine macrophages (65), may be responsible for the extended time of infections since this cytokine prevents the activation of effector mechanisms for pathogen destruction (233, 237).

## Perspectives

The economic loss from *U. diversum* infection is reflected in bovine meat, milk, and semen production and marketing industries. In veterinary research, PCR technology for *U. diversum* diagnosis is a new paradigm for detecting this infectious agent. The early, rapid diagnosis and control of bovine ureaplasmal infections must be consolidated soon to reduce the costs in keeping cattle healthy.

The sequencing of the *U. diversum* genome has provided more information about the species, especially regarding the antigenic variation (28, 90). Now CDS for various LAMPs, VSPs, and other antigens are available. An alternative that seems promising for future studies would be to review the heterologous expression of these antigens by recombinant DNA technology, immunomodulation, and antibody production assays.

Studies of *U. diversum* LAMPs showed their potential to increase the host proinflammatory cytokines (42). However, molecular cloning and expression in heterologous systems of these proteins should provide a better understanding of this ureaplasma virulence. However, the culturing techniques for *U. diversum* must also be improved, as the high nutritional requirements make laboratory isolation and cultivation difficult. This aspect, added to the high specificity of ureaplasmas to host tissues, also limits studies of pathogen-host interaction. This limitation has been solved in other fastidious microorganisms through the cultures of organoids from target tissues of infection (238). However, use of this technology is not yet a reality for studying mollicutes. The heterologous protein expression in *Escherichia coli* is already known and this should facilitate more specific studies. The other approach should include the ability to better understand the effects of purified antigens. These technologies have already been applied in other *Mollicutes* and should help improve understanding of the pathogenesis and virulence of bovine ureaplasma.

## Author Contributions

MS, JT, and LM: conceived and designed. MS, NM, GC, BB, JT, and LM: wrote the paper. All authors: read and approved the final manuscript.

## Conflict of Interest

The authors declare that the research was conducted in the absence of any commercial or financial relationships that could be construed as a potential conflict of interest.
